# An easy “SteamDrop” method for high quality plant chromosome preparation

**DOI:** 10.1186/1755-8166-7-21

**Published:** 2014-03-06

**Authors:** Ilya Kirov, Mikhail Divashuk, Katrijn Van Laere, Alexander Soloviev, Ludmila Khrustaleva

**Affiliations:** 1 Department of Genetics and Biotechnology, Russian State Agrarian University-MTAA, Timiryazevskay str. 49, 127550 Moscow, Russia; 2 Center of Molecular Biotechnology, Russian State Agrarian University-MTAA, Listvennichnaya Alleya 5, 127550 Moscow, Russia; 3 Institute for Agricultural and Fisheries Research (ILVO), Plant Sciences Unit, Applied Genetics and Breeding, Caritasstraat 21, 9090 Melle, Belgium

**Keywords:** Plant chromosome preparation, Fluorescence in situ hybridization, Steam application, New method

## Abstract

**Background:**

The chromosome preparation is a crucial step for obtaining satisfactory results in molecular cytogenetic researches. The preparation of plant chromosomes for molecular cytogenetic purposes remains a challenge for some species. In contrast to human chromosome preparation, the processes occurring during plant chromosome preparation and causing chromosome spreading are still poorly understood.

**Results:**

We studied the dynamics of plant chromosome spreading after dropping cell suspension on slides. We showed that steam stimulates cytoplasm hydrolysis and rapid chromosome spreading and that chromosomes stretch during this chromosome spreading. Based on these observations, we developed a novel method, named “SteamDrop”, for the preparation of well-spread mitotic and pachytene chromosomes and successfully used it for 28 plant species with large and small chromosomes. We applied cell suspensions in ethanol instead of the commonly used ethanol/acetic acid fixative. Mitotic and meiotic chromosomes prepared via “SteamDrop” were used in fluorescent in situ hybridization (FISH) experiments with repetitive and unique DNA probes. Long storage of cell suspensions in ethanol did not impair the quality of chromosome preparations.

**Conclusion:**

The SteamDrop procedure provides a robust and routine method for high quality plant chromosome preparations. The method can be applied for metaphase as well as pachytene chromosome preparation in wide range of species. The chromosomes prepared by SteamDrop are well suitable for repetitive and unique DNA visualization.

## Background

Chromosome preparation is a key step in all cytogenetic techniques. Most of the modern molecular cytogenetic techniques such as FISH, GISH and Tyramide-FISH require well-spread and morphologically intact chromosomes. Several reports were dedicated to elucidating the chromosome spreading dynamic for improving human chromosome preparations [1-8], while equivalent studies are largely lacking for plants. Difficulties in obtaining well-spread plant chromosome preparation are due to the presence of a cell wall. Moreover, because the high diversity of species possessing small or large chromosomes, low or high chromosome number and different compounds in their cytoplasm, many researches on a modification of a good chromosome preparation method are conducted. There are three main methods of plant chromosome preparation: squashing [9-11], spreading [12,13] and dropping [14-22]. A squashing method has been the common procedure for chromosome counting in plant cytogenetics during decades. Another air dry/spreading method [12] involves a cell suspension preparation which is generated directly on a slide by maceration with the tip of a needle and scattered along a slide. This method is more suitable for plants with small chromosomes. A modification of the method of Pijnacker and Ferwerda [12] was made by Fukui and Ilijima [13] for rice chromosome preparation. The air dry/spreading method was developed also for studying maize somatic chromosomes [23,24]. The drop technique was developed for human cells more than a half century ago [25]. Since then, numerous improvements of the technique and comprehensive studies of all parameters influencing the human chromosome spreading dynamics have been done [3,5,7]. The first drop method on plant chromosomes was applied to isolate protoplasts [16]. That method had several disadvantages, however: (i) a low mitotic index due to cell loss during protoplast isolation and metaphase protoplast damage under hypotonic treatment; (ii) polyploidy caused by spontaneous fusion of isolated protoplasts; (iii) labor-intensive protocol; and (iv) protoplast generation from intact plant tissue is more difficult than from cultured cells. Kato et al. [20] developed a novel air dry drop method that was based on enzymatically digested root meristems and preparation of cell suspension in a tube. The cell suspension was consecutively washed in water, 100% ethanol and acetic acid/ethanol (9:1), dropped onto glass slides in a box lined with wet paper towels and dried slowly. The method was successfully used for FISH on mitotic metaphase and pachytene chromosomes of maize [20,21,26] and on mitotic metaphase chromosomes of soybean [27,28]. However, application of this method for species with large sized chromosomes remains problematic because of a low number of non-overlapped metaphases [29].

In spite of the many protocols available for plant chromosome preparation, no robust and generally applicable method has been developed. Therefore, by studying all steps of plant chromosome preparation in detail, we developed a simple protocol “SteamDrop” for reliable chromosome preparation of mitotic and meiotic plant chromosomes. A key step in the preparation of well-spread chromosomes is the application of steam at the moment of meniscus formation over cells during fixative evaporation. The applicability of “SteamDrop”-prepared chromosomes for FISH mapping of repetitive DNA as well as individual genes has been demonstrated. The chromosome preparation allowed physical mapping of small DNA fragments of onion genes using Tyramide-FISH. The “SteamDrop” method was applied for 28 species with different chromosome size and number, belonging to 13 monocot (20 species) and 7 dicot (8 species) genera.

## Methods

### Plant material

Root meristematic cells were obtained from seedlings: *Allium cepa* (2n = 2 × =16; chromosome size), *A. fistulosum* (2n = 2 × =16), *A. schoenoprasum* (2n = 2 × =16), *A. altaicum* (2n = 2 × =16), *Linum usitatissimum* (2n = 2 × =16), *Triticum aestivum* (2n = 6 × =42), *Cannabis sativa* (2n = 2 × =20).

Root meristematic cells were obtained from intensively grown plants in greenhouse: *Allium roylei* (2n = 2 × =16), *A. wakegi* (2n = 2 × =16), *Humulus japonicus* (2n = 2 × =17 for male or 2n = 2 × =16 for female plants), *H. lupulus* (2n = 2 × =20), *Rosa wichurana* (2n = 2 × =14), *Populus nigra* (2n = 2 × =38), *Brassica oleracea* (2n = 4 × =36), *Ricinus communis* (2n = 2 × =20), *Anthurium andreanum* (2n = 2 × =30), *Monstera deliciosa* (2n = 4 × =60), *Philodendron scandens* (2n = 2 × =32), *Spathiphyllum wallisii* (2n = 2 × =30), *Syngonium auritum* (2n = 2 × =24), *Zantedeschia elliotiana* (2n = 2 × =32), *Aloe vera* (2n = 2 × =14), *Hippophae rhamnoides* (2n = 2 × =24), *Festuca arundinacea* (2n = 6 × =42) and *Lolium perenne* (2n = 2 × =14), *Thinopyrum ponticum* (2n = 10 × =70), *Th. elongatum* (2n = 2 × =14).

Shoot meristems collected from seedlings for *Triticum aestivum* and Triticale (2n = 6 × =42) or from plants in the greenhouse for *R. wichurana* were also used as a source of divided cells for chromosome preparation.

### Metaphase arresting, fixation and enzyme treatment

For pretreatment and metaphase arresting, see Table 1. After pretreatment, the roots, shoots or anthers with pollen mother cells (PMC) were fixed in 3:1 (ethanol:acetic acid) for 30–50 min at room temperature. Cell suspension were prepared strait away or cell sources were stored overnight in the freezer at −20°C preceeding enzyme treatment.

**Table 1:** Condition of metaphase arresting and enzyme treatment

**Species**	**Reagents and conditions**	**Enzyme concentration**	**Incubation time in enzyme**
*Allium cepa*, *Allium fistulosum*, *Allium schoenoprasum*, *Allium altaicum*	0.75 mM hydroxyurea for 20 h (RT), 0.05% colchicine for 3.5-4 h (RT)	0.6%	90–100 min
*Humulus japonicus*, *Humulus lupulus*, *Linum usitatissimum*, *Cannabis sativa*, *Ricinus communis*	2 mM 8-hydroxyquinoline, 4 h	0.6%	100–120 min
Triticale, *Triticum aestivum*, *Thinopyrum ponticum*, *Thinopyrum elongatum*	0.2% colchicine, 2 h^1^	1.2%	100–120 min
*Brassica nigra*, *Brassica oleracea*,	1-bromnaphtalene (1:1000 water solution) overnight 4°C	0.6%	90–100 min
*Aloe vera*,		1.2%	100–120 min
*Rosa wichurana*		0.3%	90–120 min
*Anthurium andreanum*, *Monstera deliciosa*, *Philodendron scandens*, *Spathiphyllum wallisii*, *Syngonium auritum*, *Zantedeschia elliotiana*, *Hippophae rhamnoides*, *Festuca arundinacea* and *Lolium perenne*		0.1%	60–90 min
*Rosa wichurana*^*2*^	Mix of 0.1% colchicine and 2 mM 8-hydroxyquinoline (4 h, RT)^3^	1.2%	120–150 min
*Allium wakegi*, *Allium roylei*	Nitrous oxide gas (1.0 MPa), 3 h	0.6%	90–100 min
*Allium cepa* PMC	-	1.5%	180–200 min

^1^the same treatment was used for shoot meristems of Triticale and *Triticum aestivum*.

^2^applied to shoot meristems.

^3^this procedure was carried out according to Ma et al., 1996 [30].

The stock enzyme mixture, containing (w/v) 6% Pectolyase Y-23 (Kikkoman, Tokyo, Japan), 6% Cellulase Onozuka R-10 (Yakult Co. Ltd., Tokyo, Japan) and 6% Cytohelicase (Sigma-Aldish Co.LLC, France), was prepared in 0.1 M citric buffer (pH4.8). Concentrations of the work enzyme mixture and incubation time for different species are listed in Table 1.

### Protocol of chromosome preparation using the “SteamDrop” method

#### *Enzyme treatment*

1. Wash roots (anthers or shoots) in water for 10–30 min

2. Dissect meristems and transfer them into 0.1M citric buffer, pH 4.8

3. Transfer 1–5 meristems to 0.5 ml tubes with 20–30 μl of enzyme mixture (Table 1)

4. Incubate at 37°C for 1–2.5 h depending on species (Table 1)

#### *Cell suspension preparation*

1. Vortex the tubes with digested meristems to get cell suspension

2. Add 600 μl of distilled water and mix

3. Centrifuge at 10,000 rpm for 45 sec

4. Remove supernatant using a Pasteur pipette

5. Add 600 μl of 96% ethanol and mix (cell suspension can be stored at −20°C for at least 6 months)

6. Centrifuge at 11,000 rpm for 30 sec

7. Discard supernatant by inverting the tube

8. Resuspend the pellet in 20–100 μl of 96% ethanol, depending on cell concentration.

#### *Chromosome preparation*

1. Drop 10 μl of cell suspension onto a slide* and wait till the surface becomes granule-like, i.e. ethanol meniscus occurred on the top of the cells, (10–15 sec)

2. Drop 18–22 μl of fixative (1:1, 2:1, 3:1 or 5:1 ethanol:acetic acid)** and wait till the surface becomes granule-like and the layer of fixative becomes thin (25–35 sec)

3. Put the slides upside down under the steam from a water bath at 55°C (10–15 cm from water surface of the water bath) for 3–5 sec

4. For double “SteamDrop”, repeat step 2 but with less volume (3–6 μl) of fixative and higher concentration of acetic acid. Perform Step 3 for 1 sec only.

5. Immediately dry slides with air flow (e.g. a tabletop fan).

Note

*-for preparation of large size chromosomes it is useful to coat slides with APES (3-aminopropyl-triethoxy-silane) to prevent a chromosome partial detachment. APES coating of slides: 1.5% APES in 100% acetone for 30 sec, twice wash in distilled water and dry for 1 h at 37°C.

**– the protocol allows an easy correction of enzyme treatment results - check the level of tissue enzymatic digestion in the first chromosome preparation slide under microscope, if tissue is underdigested use a high proportion of acetic acid in fixative (1:1 or 2:1); if tissue is overdigested use a low proportion of acetic acid in fixative (5:1 or 10:1).

### Probe preparation

#### *LFS and bulb alliinase gene fragment*

The LFS and bulb alliinase gene fragment probes were obtained using specific primers (for LFS: LFSbeF: 5′-AAATGGAGCTAAATCCTGGTG-3′, LFSbeR: 5′-CATAATGCATCACAGCACTGAA-3′; for alliinase: Allbe1F: 5′-GGTCATCTCCCTTTCACCAA-3′, Allbe1R: 5′-TGATCAAACTCAAACGCAC-3′) designed by Primer 3.0 software (http://frodo.wi.mit.edu/) using LFS [GenBank: AB094593.1] and alliinase [GenBank: L48614] sequences from GenBank at the NCBI (http://www.ncbi.nlm.nih.gov/genbank/). The PCR conditions were 94°C – 1 min, 35 cycles; 94°C – 1 min; 58°C – 1 min; 72°C – 1 min; final elongation: 72°C – 3 min. The PCR products were cloned by pPCR-TOPO kit (Invitrogen, Carlsbad, California, USA) according to the manufacturer’s descriptions. Plasmid DNA was isolated from white colonies, sequenced and sequences were analyzed by BLASTN. Plasmids with high similarity to LFS gene or to bulb alliinase gene fragment were selected for labeling with the Biotin Nick Translation Mix (Roche Diagnostics Gmbh, Mannheim, Germany).

#### 5S rDNA

A plasmid carrying the 5S rRNA gene of rye (pScT7, [31] was labeled by Biotin-16-dUTP using Biotin- Nick Translation Mix according to the manufacturer’s protocol (Roche Diagnostics Gmbh, Mannheim, Germany).

#### *HJSR KpnI*

A plasmid carrying a HJSR *Kpn*I repeat of *Humulus japonicus *[32] was labeled by Digoxigenin-11-dUTP using Digoxigenin - Nick Translation Mix according to the manufacturer’s protocol (Roche Diagnostics Gmbh, Mannheim, Germany).

#### (AAC)_5_ oligonucleotide

(AAC)_5_ oligonucleotide labeled with biotin in the 3′- and 5′ -ends was synthesized in ZAO ‘Syntol’ (Moscow, Russia).

### DNA isolation

Genomic DNA was isolated according to Rogers and Bendich [33].

### Tyramide-FISH

Probe hybridization and signal detection was performed according to Khrustaleva and Kik [34] with minor modifications. Before the RNAse treatment and denaturation step, slides were fixed in 4% paraformaldehyde buffered in 1 × PBS (10 × PBS: 1.3 M NaCl, 70 mM Na_2_HPO_4_, 30 mM NaH_2_PO_4_, pH 7.5) for 8 min and 10 min, respectively. The step of endogenous peroxidases inactivation was carried out by exposing the slides to 0.01 M HCl for 8 min. The hybridization mixture consisted of 50% (v/v) deionized formamide, 10% (w/v) dextran sulphate, 2 × SSC, 0.25% sodium dodecyl sulphate, 2.75 ± 1.00 ng/μl probe DNA. The mixture was denatured at 75°C for 5 min and subsequently placed on ice for 5 min. Sixty microliters of the mixture was added to the chromosome preparations, covered with a coverslip (22 × 32 mm), and denaturated for 5 min at 80°C. An 82% stringency washing was applied: slides were washed in 2 × SSC twice for 5 min at 37°C, in 25% (v/v) formamide in 0.4 × SSC twice for 10 min at 42°C, then in 2 × SSC for 3 min at 37°C. The tyramide detection solution was prepared by thoroughly mixing a 1:50 tyramide-Fluorescein stock solution in amplification diluent (Perkin Elmer, Inc., Waltham, Massachusetts, USA) with 10% (w/v) dextran sulfate.

### FISH

FISH procedure with 5S rDNA and biotinylated (AAC)_5_ as probes was carried out according to the protocol of Heslop-Harrison et al. [35] and Schmidt et al. [36] with slight modification of the slide preatreatment before adding the hybridization mix. Additional treatment with 4% buffered paraformaldehyde solution (BPS), pH 8.0, for 9 min before RNAse treatment was used and pepsin treatment was excluded.

### Microscopy and image analysis

Slides were examined under a Zeiss Axio Imager microscope (Carl Zeiss MicroImaging, Jena, Germany). Selected images were captured using an Axio Cam MRm digital camera. Image processing and thresholding were performed using AxioVision ver.4.6 software (Carl Zeiss MicroImaging, Jena, Germany). Final image optimization was performed using Photoshop (Adobe Inc., San Jose, California, USA).

## Results

### Dynamics of chromosome spreading under steam

The experiment has been done using *Allium cepa*, a species with large chromosomes, and *Humulus japonicus*, a species with small chromosomes. Morphological changes of metaphase chromosomes during slide preparation under 50% relative humidity (RH) and room temperature (25°C) were observed. Phase-contrast microscopy was used to visualize chromosome structure with high resolution and to check the amount of remaining cytoplasm. We analyzed the dynamics of chromosome spreading in both the standard procedure and the proposed steam application procedure (“SteamDrop”).

**Figure 1:**
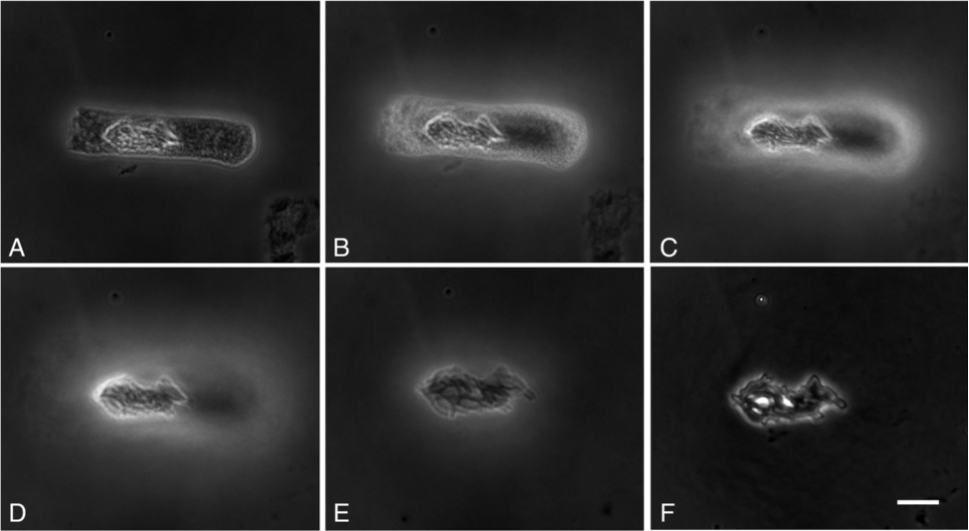
**Dynamics of morphological changes of *A. cepa *metaphase chromosomes in the standard protocol.** The meniscus formation **(A-B)** and a slight cell swelling **(C-F)** during fixative evaporation from the slide surface. Bar = 10 μm.

Using the standard protocol, a drop of cell suspension in ethanol:acetic acid fixative (3:1) was placed on the slide. During fixative evaporation (15–25 seconds) the meniscus formation (Figure 1A-B), cytoplasm hydrolysis (Figure 1C-D) and a slight cell swelling (Figure 1D-F) occurred. In most metaphases the chromosomes remained close together.

**Figure 2:**
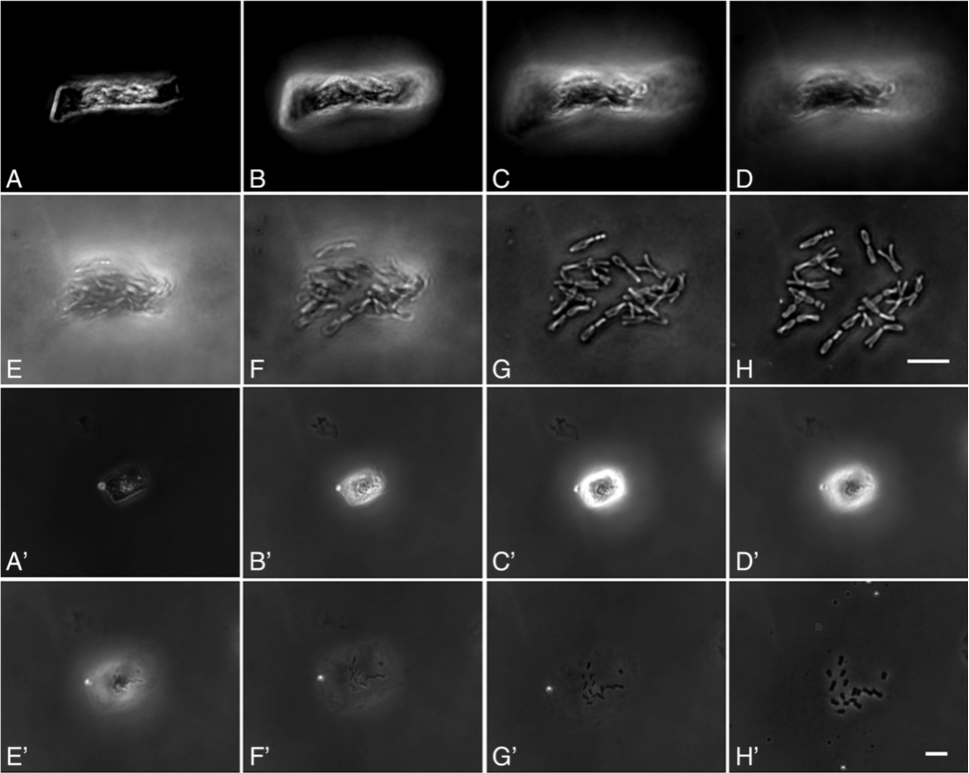
**Dynamics of *Allium cepa *(A–H) and *Humulus japonicus *(A’-H’) chromosome spreading understeam.** Meniscus formation **(A-B, A’- C’)**, cell swelling **(C-D, D’-E’)** and chromosome spreading **(E–H, F’-H’)**. **C–H** and **D’-H’** - steam application to the slides. Arrows indicate stretching of an *Humulus japonicus* chromosome. Bar = 10 μm.

Using the “SteamDrop” method a drop of cell suspension in 96% ethanol was placed on the slide. When the surface became granule-like, a drop of fixative (3:1 ethanol:acetic acid) was added. During the next 25–35 seconds, the fixative evaporated and the granule-like surface again appeared (Figure 2A-B, 2A’-C’). The moment of granule-like surface appearance after fixative addition was the crucial stage for steam application and obtaining good chromosome spreading. The stage under influence of steam was the shortest stage, lasting only few (3–5) seconds. During this stage, cell swelling, fast chromosome spreading and hydrolysis of the cytoplasm occurred (Figure 2C–H, 2D’-H’).

### Slide drying conditions

The next step after steam exposure was slide drying. To check the influence of the slide drying condition on the chromosome spreading, we compared drying (1) at natural condition (50% RH, 22–25°C) and (2) with additional air flow using a fan. In the first experiment the drying process was slow (20–30 sec), resulting in a high number of ‘overspread’ metaphase plates with chromosome loss. Under air flow conditions, the slides dried faster, causing reduced overspreading and as consequence the preservation of complete chromosome sets in metaphases. Also microscopic observations of chromosome behavior during the drying process without intensified air-flow showed that chromosomes and whole cells floated away with fixative currents. The phenomenon became weaker when air-flow drying hastened the evaporation of fixative from the slide. Thus, cells kept their positions on the slide.

### Effect of relative humidity on chromosome spreading and chromosome length

To estimate the effect of the relative humidity on chromosome spreading, different RH (25–30%, 50–55% and 65–70%) at room temperature (22–25°C) were applied. *Allium cepa* was used for this experiment. Adjustment of RH in an isolated room-box was done manually. The RH was measured by hygrometer Testo 625 (Testo AG, Lenzkirch, Germany).

Microscopic observation showed that under moderate RH (50–55%), the chromosome spreading was going on during the whole period of steam exposure, resulting in up to 60% of well spread chromosomes without cytoplasm. Under high RH (65–70%) in most cases the chromosomes were clumped, did not spread well after steam exposure and cytoplasm hydrolysis was often incomplete. Preparation under low RH (25–30%) resulted in early cytoplasm hydrolysis that prevented chromosome spreading.

**Figure 3:**
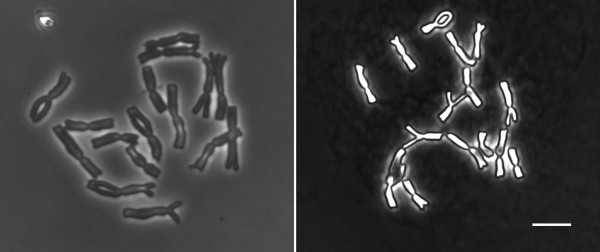
**Metaphase chromosomes of *Allium cepa *prepared under moderate (50%) RH (left) and high (70%) RH (right).** Bar = 10 μm.

The total chromosome length has been measured on slides prepared under moderate or high RH using a single cell suspension sample. The chromosomes prepared under high humidity were significantly smaller (163 ± 14 μm) than chromosomes prepared under moderate humidity (232 ± 1.0 μm, P ≤ 0.0001). Under high RH the total chromosome length had a wide range, while under moderate RH it was almost uniform. The chromosomes prepared under high RH appeared as bright light structures of high optical density under a phase contrast microscope, while the chromosomes prepared under moderate RH were less condensed and appeared as grey structures (Figure 3).

### Chromosome spreading using “SteamDrop”

**Figure 4:**
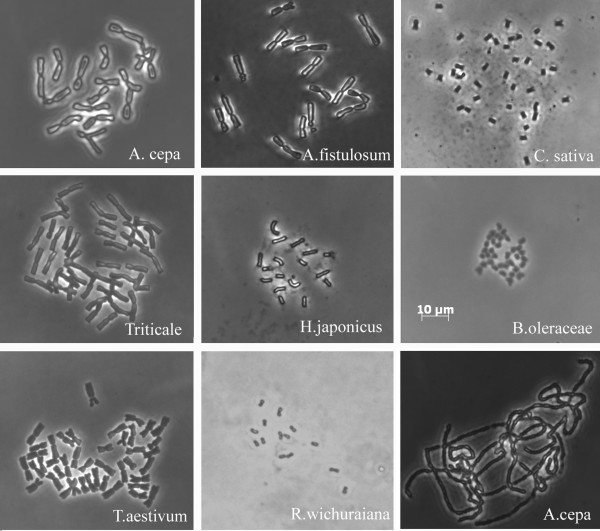
**Mitotic metaphase plates of species with large (*Allium cepa, Allium fistulosum, *Triticale*, Triticum aestivum*) and small (*Cannabis sativa*, *Humulus japonicus, Brassica oleracea, Rosa wichurana*) chromosomes.** The last figure is pachytene chromosomes of *A. cepa*. All photos were made using the same magnification. Bar = 10 μm.

In species with small chromosomes, most of the metaphase cells (85–97%) prepared with the “SteamDrop” method showed good chromosome spreading (<2 overlapped chromosomes, Figure 4 *Cannabis sativa*, *Humulus japonicus*, *Brassica oleracea*, *Rosa wichurana*). In species with large chromosomes only 15–20% of metaphases appeared well spread. Therefore, for these species a second drop of fixative with higher acetic acid concentration (e.g. 1:1 or glacial acetic acid) after the first drop of 5:1 or 3:1 ethanol:acetic acid were used (Figure 4 *Allium cepa*, *Allium fistulosum*, Triticale, *Triticum aestivum*). The second drop, containing a higher portion of acetic acid, completed the cytoplasm digestion and provided additional chromosome spreading before chromosome immobilization on the slide surface. Steam was applied after each drop of fixative. Microscopic analysis of chromosome spreading revealed that the use of two drops of fixative extended the time of chromosome spreading. Using a second fixative drop in onion increased the number of well spread metaphases two-fold as compared to a single drop treatment. It should be mentioned that the application of nitrous oxide gas as metaphase arresting agent in combination with the second fixative drop increased the number of well spread metaphases up to 60% in *Allium* species. Application of a second drop in species with small chromosomes (*Cannabis sativa*, *Humulus japonicus*, *Humulus lupulus*, *Linum. usitatissimum*, *Populus nigra*, *Brassica oleracea*, *Ricinus communis*) resulted in a higher percentage of overspread metaphases and chromosome loss as compared to the single drop use. However, this was not true for *Rosa wichurana*, *Spathiphyllum wallisii*, *Syngonium auritum*, *Zantedeschia elliotiana*. These species have also small chromosomes but a rigid cell wall and the application of the second drop resulted in well spread metaphases with completely digested cytoplasm.

The application of the second drop was also successful for meiotic chromosome preparation using a higher concentration of enzymes (1.5% cellulase, 1.5% pectolyase, 1.5% cytohelicase) for 3 h and a higher concentration of acetic acid in fixative (1:1) for first drop and 100% acetic acid for second drop (Figure 4. *Allium cepa*).

### Cell suspension storage

Our experiments showed that cell suspension of PMCs and root meristems in 96% ethanol can be used for chromosome preparation even after 6 months of storage in freezer (−20°C). The storage did not influence the chromosome preparation quality. Whereas, commonly used storage of anthers for several months in ethanol:acetic acid fixative or in 70% ethanol resulted in poor chromosome spreading of PMCs impaired by a high amount of cytoplasm. The “SteamDrop” method makes it possible to prepare mitotic and meiotic chromosomes independent of the season.

### “SteamDrop” chromosome preparations are excellent for FISH

Chromosomes prepared by the “SteamDrop” method were evaluated for their applicability in FISH experiments. It was found that chromosome preparations are highly sensitive to denaturation in the hybridization mixture. Immediately after denaturation, DAPI stained chromosomes sometimes showed chromatin protrusion that often hampers signal detection and karyotyping. Application of an additional treatment with 4% buffered paraformaldehyde solution (BPS), pH 8, for 9 minutes before RNAse treatment helped to overcome this problem. Furthermore, chromosome preparation according to the “SteamDrop” protocol does not require pepsin pretreatment. The “SteamDrop” preparations were largely free of cytoplasm and yielded a high signal to noise ratio. In our experience, pepsin pretreatment did not increase signal to noise ratio, but might damage chromatin structure.

**Figure 5:**
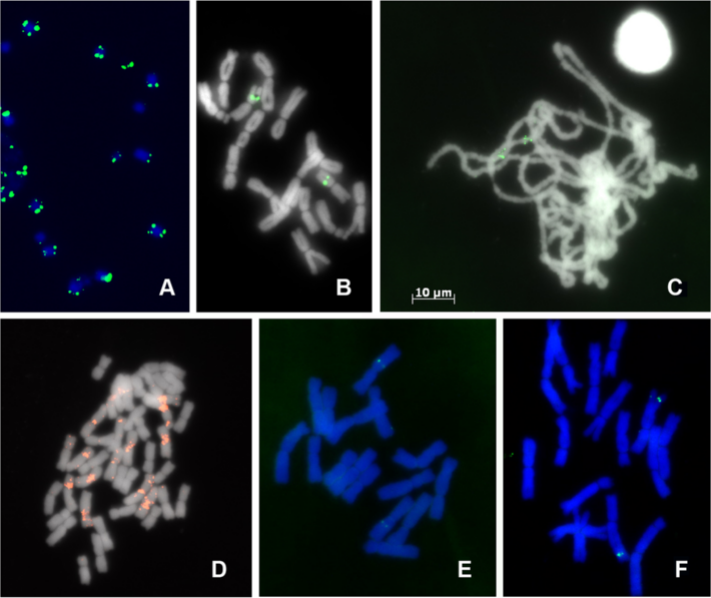
**The “SteamDrop” chromosome preparations used in different cytogenetic techniques. (A)** FISH - *Humulus japonicus* probing with the HJSR *Kpn*I repeat; **(B)** FISH - *Allium fistulosum* probing with 5S rDNA (pSCT7); **(C)** FISH - *Allium cepa* pachytene chromosomes probing with 5S rDNA (pSCT7); **(D)** FISH - *Triticum aestivum* probing with the (AAC)_5_ oligonucleotide; **(E)** Tyramide-FISH - *A. cepa* probing with the LFS gene clone (550 bp); **(F)** Tyramide-FISH - *A. cepa* probing with bulb alliinase gene fragment (1.1 Kb).

FISH experiments on mitotic chromosomes with a 5S rDNA probe in *Allium fistulosum* (Figure 5B) or HJSR *Kpn*I probe in *Humulus japonicus* (Figure 5A) or the (AAC)_5_ oligonucleotide probe in *Triticum aestivum* (Figure 5D) were analyzed. The chromosomal positions of the corresponding FISH signals coincided with results described earlier [37,32].

FISH on the pachytene chromosome of *Allium cepa* with the 5S rDNA revealed two hybridization sites (Figure 5C), in accordance with previously obtained data on mitotic chromosome of *Allium cepa *[37].

Detection of genes on plant chromosomes strongly depends on the quality of chromosome preparations. The applicability of the “SteamDrop” chromosome preparations for gene localization was evaluated in Tyramide-FISH experiments. Two genes of *Allium cepa*, the LFS (lachrymatory factor synthase) and the bulb alliinase, which are involved in the same biochemical pathway, were used for visualization on mitotic metaphase chromosomes. The LFS gene (550 bp) was detected in a proximal position on chromosome 5 (Figure 5E); the bulb alliinase gene fragment (1.1 Kb) was found in a distal position on chromosome 4 (Figure 5F), as reported previously [38,39].

## Discussion

### Steam stimulates chromosome spreading

Steam application at the moment of meniscus formation causes effective chromosome spreading. Steam hastens cell wall hydrolysis by heating the slide surface. This stimulates ethanol evaporation and increases the acetic acid concentration for cellulose hydrolysis [40]. Moreover, steam delivers water to the slide surface [5], providing rapid cell rehydration. Claussen et al. [6] showed the essential role of water in mammalian cell swelling. Kato et al. [20] also emphasized the importance of a high humidity treatment in order to spread plant chromosomes and thus proposed the use of a humidity chamber during slide preparation. We propose to use steam for efficient chromosome spreading. We suppose that during steam application three coinciding processes occur: (1) cytoplasm rehydration and swelling; (2) rapid cell wall hydrolysis, which aids cell burst due to cytoplasm swelling; (3) chromosome movement. It is very important that the steam is applied at the moment a granule-like surface appears, when meniscus formation is occurring. Meniscus pressure and steam cause rapid plant cell swelling and, as a consequence, efficient chromosome spreading.

### Effect of relative humidity

Mammalian chromosome spreading depends on relative humidity of the environment [3-7]. We found that RH influences on plant chromosome preparation as well. The optimal RH for plant chromosome preparation using our “SteamDrop” method was 50–55%, similar to that described for mammalian chromosome preparation [3]. We suppose that the impact of RH on chromosome spreading is mainly determined by slide drying time and water-induced cytoplasm swelling. Under low RH, quick ethanol evaporation with increasing acetic acid concentration occurs (fixative is not an azeotropic mixture, [6]) resulting in prompt cytoplasm digesting before steam application. Cytoplasm swelling does not take place and chromosomes remain close together. Under high RH, ethanol evaporates slowly while the process of fixative rehydration goes fast. This results in a low concentration of acetic acid. Therefore, undigested thick cytoplasm hampers steam-induced chromosome spreading. Under mid-level RH, the processes of the fixative rehydration and the ethanol evaporation are balanced. At the moment of steam application, cytoplasm density is sufficient for steam-induced chromosome spreading.

### “SteamDrop” may cause chromosome stretching

Plant chromosome stretching was observed under steam action. It was found that the degree of chromosome stretching depends on RH of ambient condition. Thus chromosomes prepared under moderate humidity were 1.42 times longer that those prepared under high RH. Claussen et al. [6] showed chromosome preparation-induced changes in the lengths of human lymphocyte chromosomes. Authors proved that chromosomes have their own potential to swell and they suggested that some modifications of chromosome proteins promote chromosome stretching. Moreover, it was shown by real-time scan force microscopy that DNA molecules may decondense and lengthen under specific micro-environmental condition [41]. Thus, chromosome stretching could be caused by DNA relaxation itself or rehydration of chromatin proteins or combination of both processes. Claussen et al. [6] proposed the concept of chromosomal region–specific protein swelling. They showed that “G-banded chromosomal regions” are involved in chromosome stretching. Unstretched human chromosomes do not show any visible GTG-banding patterns [4,6]. Reproducible G-banding patterns on plant chromosomes are difficult to obtain [42]. Hliscs et al. [4] supposed that the absence of plant chromosome stretching is what causes a lack of G-bands. Probably, plant chromosome stretching produced by the “SteamDrop” protocol will help to obtain reproducible G-banding patterns on plant chromosomes. Further experiments will be done to check this assumption.

## Conclusion

The results reported here demonstrate the effectiveness of “SteamDrop” method for high quality chromosome preparation of plant species with small and large chromosomes. The applicability of the chromosome preparation for FISH and Tyramide-FISH experiments was shown. The advantages and distinctions of our “SteamDrop” method from previously developed methods are (1) the steam application caused efficient chromosome spreading; (2) the minimization of washing steps reduce chromosome damage and cell loss; (3) instead of commonly used dropping onto the slide of cell suspension in ethanol-acetic acid fixative we propose to use cell suspension in 96% ethanol that allowed to regulate the chromosome spreading and amount of cytoplasm around chromosomes by adding ethanol-acetic acid fixative in proper ratio; (4) moreover, long-term storage of cell suspension in 96% ethanol does not impair the quality of chromosome preparation; (5) several slides can be prepared from a single root; (6) a simple protocol: the preparation of cell suspension excluding of metaphase arresting and enzyme treatment steps takes only 2–3 minutes; the chromosome slide preparation – 1 minute.

## Competing interests

The authors declare that they have no competing interests.

## Authors’ contributions

IK designed the study, performed the research and wrote the manuscript. KVL, AS and MD performed the research. LK designed and coordinated the study and wrote article. All authors read and approved the final manuscript.
